# Effect of Porcine Whole Blood Protein Hydrolysate on Slow-Twitch Muscle Fiber Expression and Mitochondrial Biogenesis via the AMPK/SIRT1 Pathway

**DOI:** 10.3390/ijms23031229

**Published:** 2022-01-22

**Authors:** Sun Woo Jin, Gi Ho Lee, Ji Yeon Kim, Chae Yeon Kim, Young Moo Choo, Whajung Cho, Eun Hee Han, Yong Pil Hwang, Yong An Kim, Hye Gwang Jeong

**Affiliations:** 1 Department of Toxicology, College of Pharmacy, Chungnam National University, Daejeon 34134, Korea; mpassword@cnu.ac.kr (S.W.J.); ghk1900@cnu.ac.kr (G.H.L.); jykim525@o.cnu.ac.kr (J.Y.K.); chaeyeon05@o.cnu.ac.kr (C.Y.K.); kimya@cnu.ac.kr (Y.A.K.); 2 Department of R&D, Jinju Bioindustry Foundation, Jinju 52839, Korea; ychoo@jbio.or.kr; 3 R&D Institute, AMINOLAB Co., Ltd., Seoul 06774, Korea; wjcho@aminolab.co.kr; 4 Drug & Disease Target Research Team, Division of Bioconvergence Analysis, Korea Basic Science Institute (KBSI), Cheongju 28119, Korea; heh4285@kbsi.re.kr; 5 Fisheries Promotion Division, Mokpo 58613, Korea; protoplast@hanmail.net

**Keywords:** porcine whole-blood protein hydrolysates, branched-chain amino acid, mitochondrial function, slow-twitch muscle fibers

## Abstract

Skeletal muscle is a heterogeneous tissue composed of a variety of functionally different fiber types. Slow-twitch type I muscle fibers are rich with mitochondria, and mitochondrial biogenesis promotes a shift towards more slow fibers. Leucine, a branched-chain amino acid (BCAA), regulates slow-twitch muscle fiber expression and mitochondrial function. The BCAA content is increased in porcine whole-blood protein hydrolysates (PWBPH) but the effect of PWBPH on muscle fiber type conversion is unknown. Supplementation with PWBPH (250 and 500 mg/kg for 5 weeks) increased time to exhaustion in the forced swimming test and the mass of the quadriceps femoris muscle but decreased the levels of blood markers of exercise-induced fatigue. PWBPH also promoted fast-twitch to slow-twitch muscle fiber conversion, elevated the levels of mitochondrial biogenesis markers (SIRT1, p-AMPK, PGC-1α, NRF1 and TFAM) and increased succinate dehydrogenase and malate dehydrogenase activities in ICR mice. Similarly, PWBPH induced markers of slow-twitch muscle fibers and mitochondrial biogenesis in C2C12 myotubes. Moreover, AMPK and SIRT1 inhibition blocked the PWBPH-induced muscle fiber type conversion in C2C12 myotubes. These results indicate that PWBPH enhances exercise performance by promoting slow-twitch muscle fiber expression and mitochondrial function via the AMPK/SIRT1 signaling pathway.

## 1. Introduction

Skeletal muscle, as a heterogeneous tissue, accounts for approximately 40% of adult human body weight and comprises a variety of functionally diverse fiber types. Skeletal muscle is composed of slow-twitch and fast-twitch fibers, which are classified according to their myosin heavy-chain (MyHC) isoform content, ATP production, oxidative metabolic capacity, and the type of innervation [1,2]. Slow-twitch fiber expressing slow MyHC (MyHC I) has a higher content of mitochondria, higher expression of oxidative enzymes, and is more resistant to fatigue, whereas fast-twitch fiber expressing fast MyHC has fewer mitochondria, lower endurance, and makes use of glycolytic metabolism [3]. Nutrients increase the proportion of slow-twitch muscle fibers [4,5].

Blood, which accounts for approximately 7% of the total weight of a pig, contains 75–80% moisture and 15–17% high-quality protein (albumin, globulin, and hemoglobin) [6,7]. Despite its potential as a bioresource, blood is disposed of due to a lack of research on possible industrial uses and a general aversion. The content of free amino acids, including branched-chain amino acids (BCAAs) such as valine (Val), isoleucine (Ile), and leucine (Leu), can be increased by the enzymatic hydrolysis of pig blood [8]. In humans, the three proteinogenic BCAAs Val, Ile, and Leu are among the nine essential amino acids (EAAs), accounting for 21% of the total protein content and 35% of the EAAs in muscle [9,10]. Leu is important in the regulation of muscle protein synthesis [11,12,13]. In C2C12 cells, Leu induces myofibrillar protein accretion and reduces the MyHC mRNA level [14]. Therefore, we hypothesized that porcine whole-blood protein hydrolysate (PWBPH) enhances exercise performance and resistance to exercise-induced fatigue by promoting the conversion of muscle fiber type, from fast-twitch to slow-twitch, and mitochondrial biogenesis.

## 2. Results

### 2.1. PWBPH Increases Endurance Performance and Relative Muscle Weight

**Figure 1:**
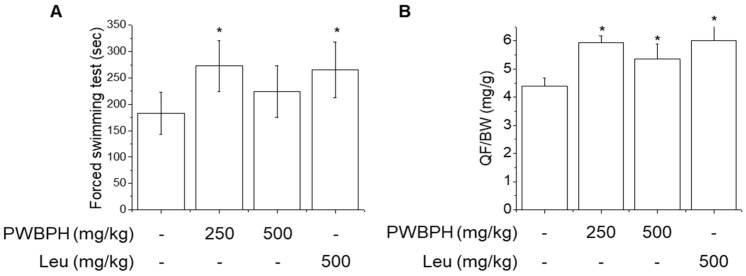
Effect of PWBPH on endurance performance and muscle weight. (**A**) Exercise function was determined based on the time to exhaustion in the forced swimming test (*n* = 6). (**B**) QF muscle/body-weight ratios in the control, PWBPH 250, 500 mg/kg, and Leu 500 mg/kg groups (*n* = 6). * *p* < 0.05 vs. control group. Abbreviations: QF, quadriceps femoris; BW, body weight).

To evaluate the effect of PWBPH supplementation on endurance capacity, we performed a forced swimming test. As shown in Figure 1A, PWBPH and Leu supplementation increased the time to exhaustion in the forced swimming test. No significant difference was found in exhaustion time between the control group and the 500 mg/kg PWBPH group. PWBPH and Leu supplementation significantly decreased the exercise-induced increases in lactate and blood urea nitrogen (BUN) levels (Table 1). PWBPH and Leu supplementation showed a tendency to decrease the level of creatine kinase (CK), and the effect of 250 mg/kg PWBPH was significant. PWBPH and Leu supplementation significantly increased the relative quadriceps femoris (QF) muscle weight compared with the control group.

A low dose (250 mg/kg) of PWBPH exerted a greater effect on endurance capacity, QF muscle gain, and anti-muscle fatigue, compared to 500 mg/kg PWBPH, suggesting that 250 mg/kg is the optimal dose in mice. Therefore, the 250 mg/kg PWBPH group was used to investigate muscle fiber type conversion.

### 2.2. PWBPH Increases Slow-Twitch Muscle Fibers and Mitochondrial Function-Related Markers In Vivo

**Figure 2:**
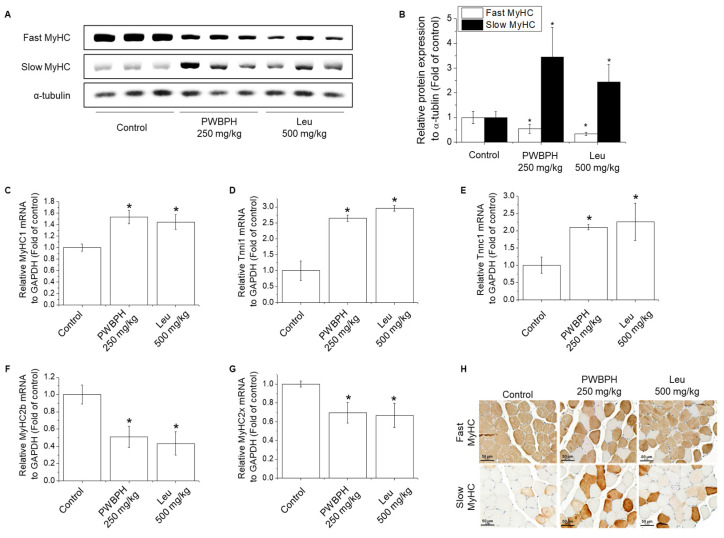
Effect of PWBPH on transformation from fast-twitch to slow-twitch muscle fiber type. (**A**) The protein levels of fast MyHC and slow MyHC in QF muscle of the control, PWBPH 250 mg/kg, and Leu 500 mg/kg groups were determined by Western blotting and (**B**) the band intensities were measured using ImageJ software. (**C**–**G**) qRT-PCR analysis of the mRNA levels of slow-twitch muscle fiber-related genes (*MyHC1*, *Tnni1*, and *Tnnc1*) and fast-twitch muscle fiber-related genes (*MyHC2b* and *MyHC2x*). * *p* < 0.05 vs. control group. (**H**) Histological images of the QF muscle stained with slow-twitch and fast-twitch MyHC antibodies are shown (200× magnification).

**Figure 3:**
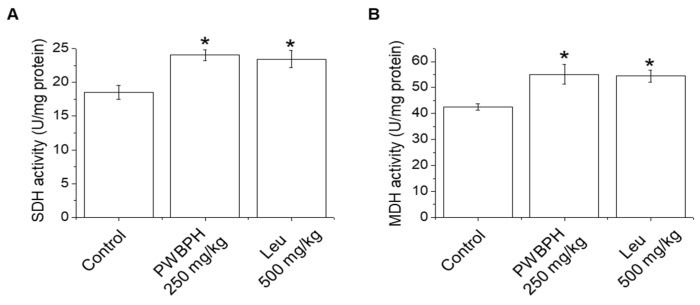
Effect of PWBPH on SDH and MDH activities. (**A**) SDH and (**B**) MDH activities in QF muscle of mice (*n* = 6) measured using commercial kits. * *p* < 0.05 vs. control group. Abbreviations: SDH, succinate dehydrogenase; MDH, malate dehydrogenase).

**Figure 4:**
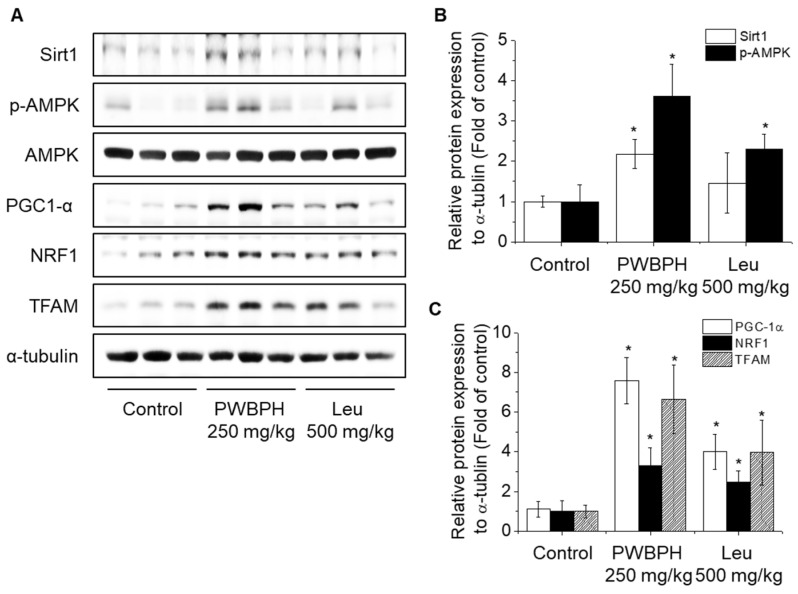
Effect of PWBPH on mitochondrial function-related protein levels in vivo. (**A**) The protein levels of SIRT1, p-AMPK, PGC-1α, NRF1, and TFAM in QF muscle of the control, PWBPH 250 mg/kg, and Leu 500 mg/kg groups were determined by Western blotting and (**B**,**C**) the band intensities were measured using ImageJ software. * *p* < 0.05 vs. control group.

We tested the effect of PWBPH and Leu supplementation on skeletal muscle fiber type conversion in mice. PWBPH and Leu supplementation significantly upregulated the slow MyHC protein level and downregulated the fast MyHC protein level compared to the control group (Figure 2A,B). Furthermore, PWBPH and Leu supplementation significantly upregulated the expression levels of genes associated with slow-twitch fibers (*MyHC1*, *Tnni1*, and *Tnnc1*), but markedly downregulated the expression levels of genes associated with fast-twitch fibers (*MyHC2b* and *MyHC2x*) (Figure 2C–G). This similar tendency of the skeletal muscle fiber type conversion was also observed in fiber levels by the MyHC immunohistological staining assay (Figure 2H). Consistently, the Succinate dehydrogenase (SDH) and malate dehydrogenase (MDH) activities in PWBPH and Leu-treated mice were markedly increased compared to the control group (Figure 3A,B). The levels of proteins implicated in mitochondrial biogenesis (PGC-1α, NRF1, and TFAM) and regulators thereof (SIRT1 and p-AMPK) were increased by PWBPH and Leu supplementation (Figure 4A–C). Together, these results indicated that PWBPH increased the proportion of slow-twitch fibers and mitochondrial biogenesis in mice.

### 2.3. PWBPH Increases Slow-Twitch Muscle Fibers and Mitochondrial Function-Related Markers in C2C12 Myotubes

**Figure 5:**
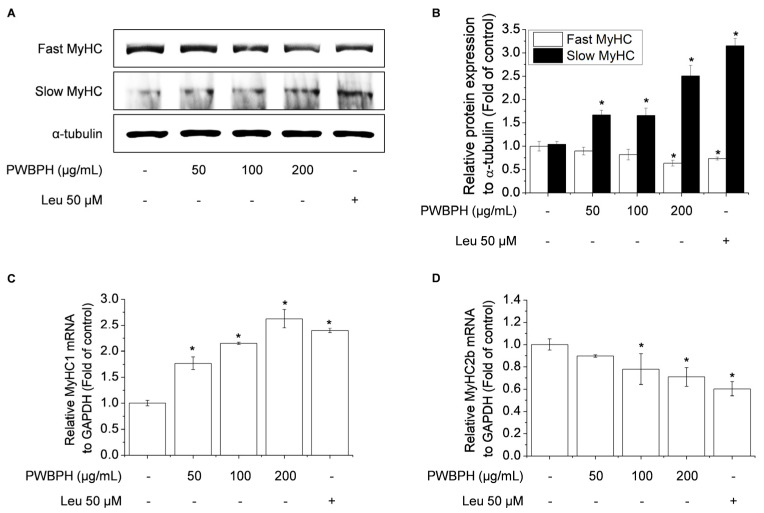
Effect of PWBPH on fast-twitch and slow-twitch muscle fiber-related protein and mRNA levels in C2C12 myotubes. About 90% confluent C2C12 cells cultured in differentiation medium for 4 days and then cells were treated with PWBPH (50, 100, or 200 μg/mL) and Leu (50 μM) for 3 days. (**A**) The protein levels of fast MyHC and slow MyHC were determined by Western blotting and (**B**) the band intensities were measured using ImageJ software. (**C**,**D**) qRT-PCR analysis of the mRNA levels of MyHC1 and MyHC2b. Data are the means ± standard deviation (*n* = 3). * *p* < 0.05 vs. control group.

**Figure 6:**
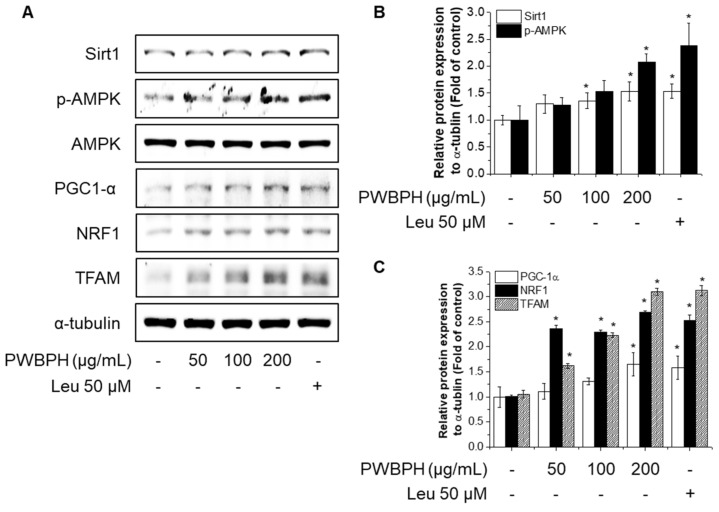
Effect of PWBPH on mitochondrial function-related protein expression in C2C12 myotubes. About 90% confluent C2C12 cells cultured in differentiation medium for 4 days and then cells were treated with PWBPH (50, 100, or 200 μg/mL) and Leu (50 μM) for 3 days. (**A**) The protein levels of SIRT1, p-AMPK, PGC-1α, NRF1, and TFAM were determined by Western blotting and (**B**,**C**) the band intensities were measured using ImageJ software. * *p* < 0.05 vs. control group.

To investigate the effect of PWBPH on muscle fiber type conversion and mitochondrial function in vitro, C2C12 myoblasts were grown to 90% confluence and replaced with differentiation medium (DM) for 4 days, and then cells were treated with PWBPH (50, 100, and 200 μg/mL) and Leu for 3 days. PWBPH decreased the fast-twitch MyHC level and increased the slow MyHC level in a concentration-dependent manner (Figure 5A,B). Consistently, PWBPH and Leu increased the MyHC1and MyHC2b gene expression level (Figure 5C,D). The levels of proteins implicated in mitochondrial biogenesis (PGC-1α, NRF1, and TFAM) and regulators thereof (SIRT1 and p-AMPK) were also concentration-dependently increased by PWBPH (Figure 6A–C).

### 2.4. Inhibition of AMPK/SIRT1 Signaling Blocks the Effect of PWBPH on Slow-Twitch Muscle Fiber-Related Gene Expression

**Figure 7:**
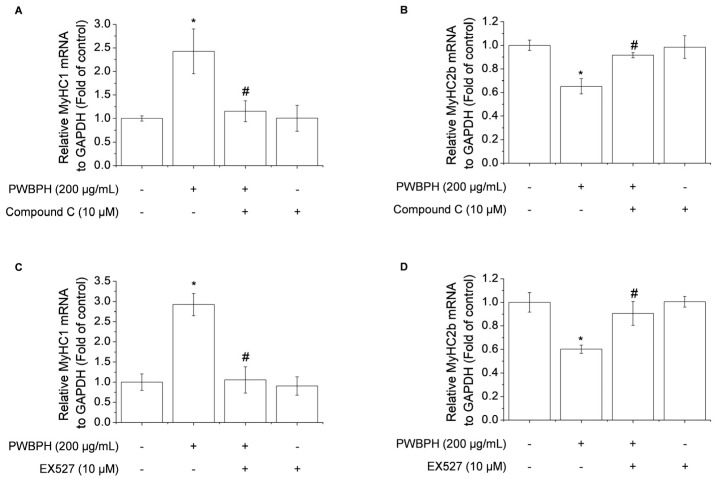
Role of AMPK and SIRT1 in PWBPH-mediated muscle fiber conversion in C2C12 myotubes. About 90% confluent C2C12 cells cultured in differentiation medium for 4 days and then cells were pretreated with the (**A**,**B**) AMPK inhibitor compound C (10 μM) or (**C**,**D**) SIRT1 inhibitor EX527 (10 μM) for 1 h before being treated with PWBPH (200 μg/mL) for 3 days. qRT-PCR analysis of the mRNA levels of *MyHC1* and *MyHC2b*. Data are the means ± standard deviation (*n* = 3). * *p* < 0.05 vs. control group. # *p* < 0.05 vs. PWBPH-treated group.

To determine whether AMPK/SIRT1 signaling is required for the promotion by PWBPH of slow-twitch muscle fibers and mitochondrial function-related gene expression, AMPK/SIRT1 signaling was blocked using the AMPK inhibitor compound C or the SIRT1 inhibitor EX527. Compound C abolished the upregulation of *MyHC1a* and downregulation of *MyHC2b* by PWBPH (Figure 7A,B). Consistently, EX527 blocked the upregulation by PWBPH of slow-twitch muscle fiber-related gene expression (Figure 7C,D). These results implicate AMPK and SIRT1 in the effect of PWBPH on slow-twitch muscle fibers.

## 3. Discussion

BCAAs are the most abundant EAAs and participate in protein synthesis and in recovery from high-intensity exercise [15,16]. Leu promotes slow-twitch skeletal muscle fiber expression in porcine skeletal muscle satellite cells. The enzymatic hydrolysis of porcine blood waste significantly increases the levels of free amino acids and bioactive peptides, including BCAAs [8,17,18,19]. However, the effect of PWBPH on muscle fiber type conversion was unclear. We showed that PWBPH supplementation significantly increased endurance capacity and QF muscle mass, and exerted an anti-muscle fatigue effect. Notably, PWBPH at 250 mg/kg (lower dose) showed the greatest effects. BCAAs exert weaker effects on biomarkers of muscle gain in mice and humans than do mixtures of BCAAs and other amino acids [20,21,22]. Therefore, PWBPH at the optimal dose enhances exercise function, and its effect is not dependent on the quantities of BCAAs.

Skeletal muscle fibers can be subdivided into slow-twitch and fast-twitch fibers according to their metabolic strategies and contraction strength. Slow-twitch fibers have higher levels of mitochondria and myoglobin, greater aerobic endurance, and a slower contractile speed than fast-twitch fibers [23]. PWBPH increased the slow MyHC protein and mRNA levels and decreased those of fast MyHC, in vivo and in vitro. Therefore, PWBPH increased the proportion of slow-twitch muscle fibers by promoting the transformation from fast-twitch to slow-twitch fibers. Skeletal muscle fiber type transformation is regulated by the metabolic enzyme activities of skeletal muscle fibers [24]. SDH and MDH are associated with the proportion of slow-twitch fibers [25]. The SDH and MDH activities were increased in QF muscle of PWBPH-supplemented mice, indicating that PHWBPH enhances skeletal muscle fiber type transformation from fast-twitch to slow-twitch.

Mitochondria are essential for ATP generation in contracting skeletal muscles [26]. PGC-1α, NRF1, and TFAM are associated with mitochondrial biogenesis [27]. PGC-1α is a key regulator of mitochondrial biogenesis and function, and AMPK and SIRT1 promote PGC-1α activity [28,29]. AMPK regulates skeletal muscle function and is phosphorylated by various nutrients [30,31]. SIRT1, which is downstream of AMPK, induces PGC-1α activity [32,33]. PGC-1α increases the protein levels of slow-twitch fibers [34]. Regular exercise training in rats induced mitochondrial protein expression and biogenesis [35]. 

In this study, PWBPH significantly increased the protein levels of p-AMPK and SIRT1 in vivo and in vitro. The inhibition of AMPK and SIRT1 attenuated the effect of PWBPH on slow-twitch muscle fiber expression. Leu reportedly increases the protein levels of phospho-AMPK and SIRT1 in porcine skeletal muscle satellite cells [36]. Leu has also been reported to increase mitochondrial content and mitochondrial biogenesis-related gene expression via SIRT1 activity and AMPK phosphorylation in C2C12 myotubes [33]. Therefore, PWBPH promotes slow-twitch muscle fiber transformation by activating the AMPK/SIRT1 signaling pathway.

## 4. Materials and Methods

### 4.1. Chemicals and Reagents

PWBPH was provided by AminoLab Co., Ltd., (Seoul, Korea). Briefly, coagulated blood was collected from a slaughterhouse and crushed using a liquefier (AminoLab Co., Ltd.); it was then treated with 1.2% food-grade serine protease (AminoLab Co., Ltd.) and hydrolyzed at 55 °C for 16 h. After hydrolysis, the blood was incubated with 5% food-grade activated carbon powder with shaking for 3 h. The hydrolysate was passed three times through filters of diatomaceous earth powder. Hydrolysis and filtration were repeated three to four times until a clear yellow liquid was obtained. The filtrate was sterilized at 85 °C for 30 min and powdered using a spray dryer. The amino acid contents were determined by the method of Hwang et al. and are presented in Table S1 [37]. Dulbecco’s modified Eagle’s medium, fetal bovine serum, horse serum, penicillin–streptomycin, and trypsin were purchased from Welgene (Gyeongsan, Korea). Antibodies against slow-twitch and fast-twitch MyHC were obtained from Sigma (St Louis, MO, USA). SIRT1, p-AMPK, AMPK, PGC-1α, NRF1, TFAM, and α-tubulin were purchased from Cell Signaling Technology (Beverly, MA, USA). All other chemicals were of the highest grade commercially available.

### 4.2. Animals and Treatment

Eight-week-old male ICR mice were obtained from Daehan Biolink (Seoul, Korea) and acclimatized to the experimental facility for 5 days. Next, the animals were randomly divided into four groups (*n* = 6 per group). ICR mice were orally administered 250 or 500 mg/kg PWBPH and 500 mg/kg Leu. Mice were housed in a controlled environment (22–23 °C, 12/12 h light/dark cycle) in accordance with the guidelines of the Animal Ethics Committee of Chungnam National University (permission number: 202012A-CNU-191, permission received on 1st February 2021). After being euthanized, the QF muscles were collected and weighed.

### 4.3. Exercise Performance

To examine the effect of PWBPH on exercise performance, a forced swimming test was performed on day 35 after oral administration. In the forced swimming test, mice carried constant loads corresponding to 5% of their body weight while swimming to analyze endurance time, as described previously [38]. Exhausted swimming was defined as a loss of coordinated movements and failure to return to the surface within 7 s.

### 4.4. Serum Biochemistry

To evaluate the anti-fatigue effect of PWBPH, mice treated for 5 weeks with PWBPH and Leu were subjected to swimming for 90 min on the day of euthanasia (day 36 after oral administration), as described previously [39]. The blood samples were collected at 30 min and centrifuged at 3000 rpm for 10 min at 4 °C. Separated serum samples were analyzed for fatigue-related indicators. After prolonged intense exercise, skeletal muscles release lactate, BUN, and CK, which were assayed using commercial assay kits (BioVision, Inc., Milpitas, CA, USA).

### 4.5. Enzyme Assays

SDH and MDH activities in cell lysates were measured using commercial assay kits (Sigma-Aldrich, St. Louis, MO, USA). Values were normalized to the corresponding total cellular protein concentration and were expressed as units per milligram of protein. Total protein content was measured using a Coomassie Protein Assay Kit (Sigma-Aldrich, St. Louis, MO, USA).

### 4.6. Cell Culture and Treatment

C2C12 mouse myoblasts were obtained from the American Type Culture Collection (Manassas, VA, USA). Cells were cultured in DMEM and maintained at 37 °C in a humidified incubator under 5% CO_2_. C2C12 cells were cultured in growth medium (GM) containing DMEM supplemented with 10% FBS, 2 mM L-glutamine, 100 U/mL penicillin, and 100 μg/mL streptomycin up to 70% confluence. During proliferation, the cells were seeded at 2 × 10^5^/well in 6-well culture plates and grown in GM. When the cells reached approximately 90% confluence, the GM was removed and replaced with differentiation medium (DM) consisting of DMEM supplemented with 2% horse serum every 2 days for 4 days, and then cells were treated with PWBPH and Leu for 3 days. A stock solution of PWBPH was prepared in distilled water. Control cells were treated with distilled water only.

### 4.7. Western Blotting

Following treatment, isolated QF muscle and C2C12 cells were lysed, and protein concentrations were measured using a protein assay kit (iNtRON, Biotechnology, Inc., Seongnam, Korea). The lysates were boiled and separated by 10% SDS-PAGE. Proteins were transferred onto PVDF membranes, which were then blocked with 5% skim milk for 1 h and incubated with primary antibodies for 3 h. The primary antibodies were detected with an HRP-conjugated secondary antibody, and the protein bands were detected using an enhanced chemiluminescence detection kit (BIOFACT Inc., Daejeon, Korea). The integrated optical density of each protein band was calculated using ImageJ software (National Institutes of Health, Bethesda, MD, USA). Values were normalized relative to the housekeeping gene α-tubulin or to the total protein.

### 4.8. RNA Extraction and Quantitative Reverse Transcriptase-Polymerase Chain Reaction

Total RNA was extracted from QF muscle and C2C12 cells using RNAiso Plus Total RNA Extraction Reagent (TaKaRa, Shiga, Japan), and cDNA was synthesized using the BioFact RT Series Kit (Biofact, Daejeon, Korea). Quantitative reverse transcriptase polymerase chain reaction (qRT-PCR) was performed to analyze gene expression with continuous monitoring using Bio-Rad CFX Connect Real-Time PCR software, version 1.4.1 (Bio-Rad Laboratories, Hercules, CA, USA). The gene-specific primer set (*MyHC1*, *Tnni1*, *Tnnc1*, *MyHC2b*, *MyHC2x*, and *GAPDH*) and protocols were from a prior study [40]. Gene expression levels were normalized to that of *GAPDH*.

### 4.9. Myosin Heavy-Chain Immunohistochemical Staining

Immunohistochemical staining assay for MyHC isoforms was performed by the modified procedure, as described previously [41]. Briefly, QF muscles were collected and immediately fixed in 10% formalin. The fixed tissue samples were embedded in paraffin and cut into transverse 10 μm thick sections. The primary antibodies were slow-twitch and fast-twitch MyHC antibodies. The expression of myosin heavy-chain isoforms on sections by the indirect immunoperoxidase method was used for muscle fiber phenotyping, and examined under a light microscope equipped with a CCD camera (BX-51; Olympus, Tokyo, Japan).

### 4.10. Statistical Analysis

The data are reported as the means ± SD. Statistical significance was determined by analysis of variance (ANOVA) followed by the Tukey–Kramer test, with *p* < 0.05 as the level of significance.

## 5. Conclusions

In conclusion, PWBPH supplementation increases endurance capacity and QF muscle mass and exerts an anti-muscle fatigue effect in mice. Additionally, PWBPH promotes muscle fiber type conversion from fast-twitch to slow-twitch and increased the levels of mitochondrial biogenesis-related markers and activators in vivo and in vitro. Furthermore, the inhibition of AMPK and SIRT1 blocked the effect of PWBPH on slow-twitch muscle fiber-related gene expression in C2C12 cells. Therefore, PWBPH may increase muscle endurance and resistance to fatigue by promoting slow-twitch muscle fiber expression and mitochondrial biogenesis via the AMPK/SIRT1 pathway.

## Figures and Tables

**Table 1:** Levels of blood markers following exercise-induced muscle fatigue.

Group	Lactate (mg/dL)	BUN (mg/dL)	CK (U/L)
Control	1.30 ± 0.12	22.5 ± 2.4	1186 ± 299
PWBPH (250 mg/kg)	1.07 ± 0.13 *	19.4 ± 1.1 *	802 ± 129 *
PWBPH (500 mg/kg)	1.12 ± 0.07 *	19.6 ± 1.1 *	966 ± 103
Leu (500 mg/kg)	1.14 ± 0.07 *	20.3 ± 0.6 *	945 ± 83

Data are shown as the means ± SD. * *p* < 0.05 vs. control group. Abbreviations: BUN, blood urea nitrogen; CK, creatine kinase).

## Data Availability

The data presented in this study are available on request from the corresponding author.

## Supplementary Materials

The following are available online at https://www.mdpi.com/article/10.3390/ijms23031229/s1.
