# Supplementary material for: Effect of Porcine Whole Blood Protein Hydrolysate on Slow-Twitch Muscle Fiber Expression and Mitochondrial Biogenesis via the AMPK/SIRT1 Pathway
Source: Int J Mol Sci. 2022 Jan 22;23(3):1229. doi: 10.3390/ijms23031229 (PMC8835758; doi:10.3390/ijms23031229)
Supplement: Supplementary file 1 [file ijms-23-01229-s001.zip › ijms-1522421-supplementary.pdf]

International Journal of *Molecular Sciences*

*Supplementary Material*

**Table S1.** Physiological amino acid compositions of PWBPH.

| | List of Amino Acids | Contents (mg/100g) |
|---|---|---|
| Physiological Amino Acid | Threonine | 1197.01 |
| | Cysteine | 70.79 |
| | Tyrosine | 1111.59 |
| | Arginine | - |
| | Alanine | 2056.28 |
| | Proline | 165.18 |
| | Lysine | 1464.38 |
| | Histidine | 984.23 |
| | Isoleucine | 611.30 |
| | Leucine | 6016.50 |
| | Methionine | 401.51 |
| | Phenylalanine | 2147.77 |
| | Tryptophan | 76.33 |
| | Valine | 1738.06 |
| | Glutamic Acid | 1850.13 |
| | Aspartic Acid | 1520.94 |
| | Serine | 1226.45 |
| | Glycine | 340.33 |
